# Association Study between *BGLAP* Gene *Hind*III Polymorphism and Type 2 Diabetes Mellitus Development in Ukrainian Population

**DOI:** 10.1155/2019/9302636

**Published:** 2019-11-27

**Authors:** Yaroslav D. Chumachenko, Viktoriia Yu. Harbuzova, Alexander V. Ataman

**Affiliations:** ^1^Scientific Laboratory of Molecular Genetic Studies, Medical Institute of the Sumy State University, 40007, Ukraine; ^2^Department of Physiology and Pathophysiology with Medical Biology Course, Medical Institute of the Sumy State University, 40018, Ukraine

## Abstract

Type 2 diabetes mellitus (T2DM) belongs to the diseases with hereditary predisposition, so both environmental and genetic factors contribute to its development. Recent studies have demonstrated that the skeleton realizes systemic regulation of energy metabolism through the secretion of osteocalcin (OCN). Thus, the association analysis between *Hind*III single nucleotide polymorphism of OCN gene (*BGLAP*) promoter region and T2DM development in Ukrainian population was carried out. 153 individuals diagnosed with T2DM and 311 control individuals were enrolled in the study. The genotyping was performed using polymerase chain reaction-restriction fragment length polymorphism (PCR-RFLP) method. The lack of association between *BGLAP Hind*III single nucleotide polymorphism (SNP) and T2DM development among Ukrainians was found. Further studies with extended groups of comparison are needed to confirm the obtained results.

## 1. Introduction

Osteocalcin (OCN) is the most prevalent noncollagenous protein in bone [1]. It is exclusively produced by osteoblasts as the pre-propeptide with a length of 100 amino acids (aa) and composed of three distinct regions—signal peptide, propeptide, and mature OCN. The first one of 23 aa is located on the N-terminus and directs protein to the endoplasmic reticulum (ER). Several major events occurred in the ER: signal sequence cleavage by signal peptidase, disulfide bond formation between 23 and 29 cysteine residues, and vitamin K-dependent *γ*-carboxylation of 17, 21, and 24 glutamate residues (Glu) by gamma-glutamyl carboxylase (GGCX). Recently, it was established that a final cellular processing step of OCN is provided by proprotein convertase furin regardless of carboxylation process. Furin recognizes C-terminal motif RX(R/K)R (RPRR for Homo sapiens) which is conserved for all vertebrates and cleavages 28 aa of propeptide region. Thus, mature OCN of 49 aa with three *γ*-carboxyglutamate residues is secreted by osteoblasts into the extracellular bone matrix where its final modification takes place determining mineralization or endocrine function of the protein [2, 3].

The large amount of studies were dedicated to investigation of OCN role in mineralization process on the analogy of matrix Gla protein (MGP) which is characterized by pronounced anticalcinogenic action [2, 4]. Moreover, completely carboxylated OCN (cOCN) has the high affinity to the hydroxyapatite (HA) which is the main component of bone matrix. Detailed structure of porcine OCN and its bone recognition mechanism was proposed by Hoang et al. (2003). It was established that OCN has globular organization and composed of three *α*-helical domains (counting from N-terminus) with short unstructured region. The vast area of negative charge in *α*1 helix due to the presence of three Gla residues was found. These *γ*-carboxyglutamates and Asp30 residue (located in *α*2 helix) bind five calcium ions that were detected to be in unexpectedly regular order reminding crystalline lattice [5]. Such strict conformity of the orientation of OCN Gla residues and HA calcium ions allowed to assume that OCN initiates the HA formation. However, subsequent studies have not confirmed this hypothesis but contributed to the OCN role in detecting physiological and pathological calcification [6].

Nowadays, it is believed that OCN's crucial role is the regulation of systemic energy metabolism after its release from bone mineral matrix to circulation. It was found that insulin renders metabolic and mitogenic effects on osteoblasts through the activation of insulin receptor and insulin growth factor receptor located on the cellular surface. This leads to the activation of MAPK and PI3K/Akt signaling pathways resulting to enhanced glucose uptake and glycogen synthesis as well as changing in osteoblastic gene profile expression [7]. On the one hand, insulin promotes Runx2 activity in osteoblasts which is the important transcription factor for OCN gene. This results to the cOCN accumulation in bone matrix. On the other hand, it was shown that expression of osteoblastic protein—osteoprotegerin (OPG)—was significantly decreased after insulin treatment of osteoblast cell culture. It is known that OPG serves as decoy for receptor activator of nuclear factor *κ*-B ligand (RANKL), which activates osteoclasts through the binding with RANK receptors. Activated osteoclasts create acidic surroundings in the resorption lacunae, and cOCN loses its carboxylic groups. Such undercarboxylated OCN (unOCN) cannot be fixed by HA and releases from bone mineral matrix to systemic circulation [8, 9].

Lee et al. have provided the first *in vivo* evidences that bone regulates systemic energy metabolism through the secretion of unOCN. Thus, the increased expression of *Insulin* and *CyclinD*1 in *β*-cells as well as *Adiponectin* in adipocytes after the unOCN treatment was showed [10]. Further studies have revealed that unOCN acts through the G protein-coupled receptor family C group 6 member A (GPRC6A) receptor, localized in different tissues, particularly in the pancreas, skeletal muscles, liver, and adipocytes [11]. Pi et al. have established that selective *Gprc6a* knockout in mice *β*-cells leads to reduction of pancreatic size and *β*-cell amount as well as decreased insulin expression and secretion. Moreover, these mice are characterized by glucose intolerance but at the same time had normal insulin sensitivity [12]. Otani et al. have demonstrated that GluOCN induces adiponectin expression in adipocytes through GPRC6A activation in an ERK/CREB/PPAR*γ*-dependent manner [13]. Du et al. showed the increased SOD, catalase, and GPx gene expression in the liver after OCN treatment. Moreover, the Nrf2 activation as well as JNK inhibition by OCN, which plays an important role in protection against nonalcoholic fatty liver disease, was established [14]. Mera et al. have found the significant role of unOCN in myofibril function in adaptation during physical exercises. They showed that unOCN facilitates the uptake and utilization of fatty acids and glucose by muscle tissue. In turn, myofibrils produce IL-6 that enhances unOCN released from bone according to the feed forward loop [15]. Several studies showed that OCN stimulates glucagon-like peptide-1 (GLP-1) secretion which further increased insulin expression and improved glucose sensitivity that could be considered another possible OCN-dependent way of systemic energy metabolism regulation [16, 17].

To date, the role of OCN in diabetes mellitus (DM) and metabolic syndrome (MS) development is actively studied [18–20]. But the main limitations of such research studies are the disregard of measurement of three fractions of protein (carboxylated, undercarboxylated, and total) and the unknown vitamin K status of patients or experimental animals. In contrast, the investigation of OCN gene polymorphism impact on the development of different metabolic diseases avoids the abovementioned difficulties and makes studies more reliable. Thus, the aim of this research was to explore the association between *BGLAP Hind*III polymorphism and type 2 diabetes mellitus (T2DM) development in Ukrainian population.

## 2. Materials and Methods

### 2.1. Groups of Comparison

The present research enrolled 153 Ukrainians (75 females and 78 males; mean age ± SD64.67 ± 8.2 years) diagnosed with T2DM and 311 (106 females and 205 males; mean age 65.65 ± 12.58 years) control subjects. The final diagnosis of T2DM was established on the basis of typical symptoms (polyuria, polydipsia, polyphagia, and weight loss), fasting glucose level, and glucose tolerance test result according to the World Health Organization criteria (WHO, 1999). Healthy subjects without any carbohydrate metabolism disorders (which was confirmed by fasting plasma glucose level less than 5.6 mmol/L and 75 g oral glucose tolerance test result less than 7.8 mmol/L) and nonburdened family history of diabetes were included in the control group. All participants of the study were selected from hospital records in 5th Sumy Clinical Hospital and Sumy Regional Diagnostic Center since 2011-2019. The protocol of the study was conformed to the Declaration of Helsinki and approved by the Ethic Committee of Medical Institute of Sumy State University (number 4/02.18.09). All individuals provided the written informed consent.

### 2.2. Genotyping

Whole venous blood was used for DNA extraction by NeoPrep^100^ DNA_*Blood* kit (Neogene, Ukraine). Polymerase chain reaction-restriction fragment length polymorphism (PCR-RFLP) was carried out for genotyping. The reaction mixture on the amplification stage consisted of 5 *μ*L FastDigest Green Buffer (10X) (Thermo Scientific™, USA), 0.5 *μ*L dNTP Mix (10 mM of each deoxynucleotide) (Thermo Scientific™, USA), 0.75 U DreamTaq DNA Polymerase (5 U/*μ*L) (Thermo Scientific™, USA), 0.1 *μ*L each primer, 75-100 ng DNA, and bidistilled water to 25 *μ*L. The primer sequences and PCR conditions are indicated in Table 1. Amplification was performed by Thermocycler GeneAmp PCR System 2700 (Thermo Fisher Scientific, USA).

**Figure 1:**
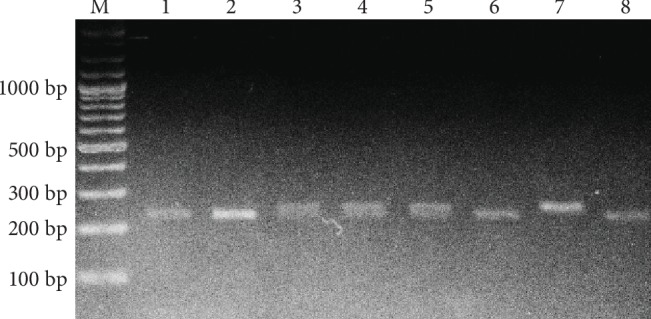
*BGLAP Hind*III polymorphism restriction analysis. M: molecular marker; bp: base pairs; lanes 1, 2, 6, 8: TT genotypes; lanes 3, 4, 5: TC genotypes; lane 7: CC genotype.

On the restriction stage, 2 U of *Hind*III (Thermo Scientific™, USA), 0.8 *μ*L of 10X Buffer R (10 mM Tris-HCl (pH 8.5), 10 mM MgCl_2_, 100 mM KCl, 0.1 mg/mL BSA) (Thermo Scientific™, USA), and bidistilled water to 2 *μ*L were added to 6 *μ*L of each sample and then incubated at 37°C for 20 hours. The presence of thymine in -198 *BGLAP* gene position leads to the cleavage of 253 base pairs (bp) amplicon into 21 bp and 232 bp fragments. The specific for *Hind*III restriction site is lost in case of thymine to cytosine replacement, and 253 bp amplicon remains undigested. Horizontal electrophoresis (10 V/cm) in 2.5% agarose gel (with 10 mg/mL ethidium bromide) and subsequent ultraviolet visualization were used for the genotype discrimination (Figure 1).

### 2.3. Statistical Analysis

The statistical analysis was done using Statistical Package for the Social Sciences software (SPSS, version 22.0, Chicago, IL, USA). Categorical variables are presented as absolute and percentage values, and continuous variables are indicated as mean ± SD with previous checking of distribution normality by the Kolmogorov-Smirnov test. The mean values of two and more groups were compared using two-tailed Student's *t*-test and ANOVA with further Bonferroni post hoc test, respectively. The accordance of allele distribution with the Hardy-Weinberg equilibrium in studied groups was detected by Calculator of Hardy-Weinberg equilibrium (https://wpcalc.com/en/equilibrium-hardy-weinberg/). The comparison of allele and genotype frequencies and all categorical variables was done by a chi-squared (*χ*^2^) test. The association between *BGLAP Hind*III and T2DM development was explored in dominant, recessive, overdominant, and additive models of inheritance using binary logistic regression. Further adjustment for age, sex, body mass index (BMI), smoking, and the presence of arterial hypertension in multivariable logistic regression contributes to increasing reliability of the obtained results. Then, logistic models with “BMI × genotype” and “sex × genotype” interaction terms were performed in order to estimate whether BMI or sex is an effect modifier, respectively. The Bonferroni correction for multiple comparisons was used to reduce the probability of type I error from occurring; thus, the obtained *P* values were multiplied by 4 (as four regression models were tested). The value *P* < 0.05 was accepted as significant.

## 3. Results

The general characteristics of comparison groups are presented in Table 2. The significant differences between BMI and fasting glucose values and systolic and pulse blood pressures (BPs) were found, as well as sex distribution and subjects with arterial hypertension and obesity (*P* < 0.05). In contrast, the T2DM and control groups were comparable by mean age (*P* = 0.379), diastolic (*P* = 0.883) and mean (*P* = 0.187) BPs, and amount of current smokers (*P* = 0.452).

The allele and genotype distributions in the T2DM and control groups are indicated in Table 3. It should be noted that genotype frequencies were corresponded to the Hardy-Weinberg equilibrium in controls (*P* = 0.888), but not in the studied group (*P* = 0.009). No significant differences between alleles and genotypes were found in comparison groups (*P* > 0.05).

The results of association analysis between *BGLAP Hind*III and T2DM development are shown in Table 4. A statistically significant dependency was found for a crude overdominant model (*P*_c_ = 0.04;OR_c_ = 0.638,95%CI = 0.415-0.979) of inheritance as well as after adjustment for age, sex, BMI, smokers, and arterial hypertension presence (*P*_a_ = 0.03;OR_a_ = 0.608,95%CI = 0.389-0.952). However, the significance of association was lost after the Bonferroni correction applying (*P*_a_^b^ = 0.12).

Then, we have prepared regression models with interaction terms in order to explore the association between *BGLAP Hind*III genotypes and T2DM development depending on sex and the presence of obesity (BMI ≥ 30 kg/m^2^). Similar results were obtained for males, females, and subjects without obesity (*P*^int^ > 0.05). In contrast, a significant association between rs1800247 and T2DM emergence was found among individuals with obesity in dominant (*P*_c_ = 0.011; OR_c_ = 0.387, 95%CI = 0.186-0.806; *P*_c_^int^ = 0.029), overdominant (*P*_c_ = 0.003; OR_c_ = 0.305, 95%CI = 0.141-0.661; *P*_c_^int^ = 0.023), and additive (*P*_c_ = 0.004; OR_c_ = 0.318, 95%CI = 0.146-0.693; *P*_c_^int^ = 0.024—for CT genotype) models. The strong protective effect of C allele was preserved after the adjustment for age, sex, smoking, and the presence of arterial hypertension in dominant (*P*_a_ = 0.018; OR_a_ = 0.399, 95%CI = 0.187-0.852; *P*_a_^int^ = 0.046), overdominant (*P*_a_ = 0.004; OR_a_ = 0.308, 95%CI = 0.138-0.687; *P*_a_^int^ = 0.038), and additive (*P*_a_ = 0.006; OR_a_ = 0.319, 95%CI = 0.142-0.717; *P*_a_^int^ = 0.037—for CT genotype) models of inheritance (Tables 5 and 6). However, after applying the Bonferroni correction, a lack of association between *BGLAP Hind*III genotypes and T2DM emergence was found in all models of inheritance (*P*^intb^ > 0.05).

Clinical characteristics of patients with T2DM stratified by genotype are demonstrated in Table 7. No statistically significant differences between *BGLAP Hind*III genotypes were found (*P* > 0.05).

## 4. Discussion

The OCN gene, bone gamma-carboxyglutamate protein (*BGLAP*) gene, is located on 1q22 chromosome with the length of 1134 bp (NC_000001.11). The thymine to cytosine transition that occurred in the -198 position of *BGLAP* promoter region (rs1800247) was discovered by Dohi et al. [21]. The scientists have published the first data regarding the distribution of *H* (22.8%) and *h* (77.2%) alleles among postmenopausal Japanese women and showed that *HH* and *Hh* genotypes had greater risk for osteopenia compared to *hh* genotype. According to 1000 Genomes Project phase 3 browser [22], the frequency of minor C allele is 0.22 in general population, 0.19 in African, 0.17 in Ad Mixed American, 0.28 in East Asian, 0.23 in European, and 0.24 in South Asian. We have established that C allele frequency in the T2DM and control groups is 20.9% and 23.0%, respectively, but the differences are not statistically significant.

Nowadays, it is known that the skeleton acts as the glucose metabolism regulator through the secretion of various osteokines, such as OCN, lipocalin 2, bone morphogenetic protein 7 (BMP7), receptor activator of nuclear factor *κ*-B ligand (RANKL), and neuropeptide Y (NPY). unOCN is considered to be an insulin secretagogue, on the one hand, as it induces *β*-cell proliferation and insulin expression, and an insulin sensitizer, on the other hand, as it promotes adiponectin upregulation and fatty acid and glucose utilization by muscle tissue [23]. Based on these facts, many research studies are devoted to association studies between OCN concentration and biochemical parameters in patients with DM and MS. Bae et al. showed that Korean postmenopausal women with MS had significantly lower concentration of serum OCN than those without MS [24]. Bao et al. established a significant association of serum OCN with MS and independent association with waist circumference and fasting plasma glucose among Chinese men [25]. Moreover, an inverse correlation between serum OCN and coronary atherosclerotic index was found. Darwish et al. demonstrated that patients with T2DM had lower cOCN and unOCN concentrations than nondiabetic individuals [26]. Moreover, the reverse association of cOCN and unOCN with BMI and body mass was found among T2DM subjects. It also should be noted that unOCN, but not cOCN, was correlated with HbA1c in the diabetic group. Takashi et al. have established the negative correlation between body fat and cOCN, as well as unOCN concentrations in patients with type 1 diabetes mellitus [27]. The link remains significant even after the adjustment for sex, diabetes duration, insulin dose, and HbA1c level. According to the meta-analysis of Liu et al., total and uncarboxylated fractions of OCN have inverse association with fasting plasma glucose and HbA1c [28].

In the present study, we have established no association between rs1800247 SNP and T2DM emergence in Ukrainian population. These results are in accordance with the previous research of Das et al. [29]. They have showed the lack of association between rs1800247 *BGLAP* and T2DM development neither in Caucasians nor in African Americans. The sizes of comparison groups were large enough to exclude *Hind*III SNP as potential T2DM risk factor. Moreover, no links were found between metabolic values as well as obesity measures and rs1800247. Ling et al. postulated that there is no significant association between rs1800247 and BMI, waist circumference, HOMA-IR, fasting insulin, fasting glucose, oral glucose test, and any lipid fractions. But they have found the protective effect of C allele against hypertension development and the reduced diastolic blood pressure in CC and TC individuals. Moreover, the significant association between arterial hypertension and rs1800247 due to SNP with HOMA-IR interaction was shown. The carriers of C allele have a decreased risk of hypertension in the subgroup with HOMA-IR less than 1.93, and no link was found among subjects with greater values [30].

In our study, we have also investigated the interaction effects between rs1800247 genotypes and other covariates in T2DM development. Thus, the protective influence of minor C allele (in the dominant model) and TC genotype (in the overdominant and additive models) against T2DM emergence among patients with obesity was shown, and this association remained statistically significant after adjustment for age, sex, smoking, and arterial hypertension presence. However, the link was lost in all models of inheritance after the Bonferroni correction adjustment. Thus, neither obesity nor gender was detected as effect modifier in the association between *Hind*III SNP of *BGLAP* promoter region and T2DM development among Ukrainians.

Several limitations of this study should be noted. First of all, the small size of comparison groups requires increasing the amount of participants to get the results that are more reliable. Then, it is necessary to find out the functional significance of *Hind*III SNP and to explore the *BGLAP* expression rates depending on the T or C presence in promoter region. In addition, it would be interesting to value OCN fractions and to investigate the appropriate correlations between genotypes and OCN concentrations.

## 5. Conclusions

In the present study, we have explored the association between *BGLAP Hind*III and T2DM development in Ukrainian population. There was no association between rs1800247 SNP and T2DM emergence in Ukrainian population. Further studies are necessary to expand the comparison groups and reveal the functional significance of SNP.

## Figures and Tables

**Table 1:** Primer sequences and PCR conditions.

Primer sequence	PCR conditions (*n* = 30 cycles)			Amplicon length	Restriction fragments
	D	H	E		
Fwd: 5′CCGCAGCTCCCAACCACAATAAGCT3′	94°C for 30 s	56°C for 60 s	72°C for 60 s	253	TT 232; 21
					TC 253; 232; 21
Rev: 5′CAATAGGGCGAGGAGT3′					
					CC 253

PCR: polymerase chain reaction; D: denaturation; H: hybridization; E: elongation; Fwd: forward primer; Rev: reverse primer.

**Table 2:** Clinical characteristics of study population.

Characteristics	T2DM	Control	*P*
	(*n* = 153)	(*n* = 311)	
Age (years)	64.67 ± 8.2	65.65 ± 12.58	0.379
Sex, female/male	75/78	106/205	0.002
BMI (kg/m^2^)	29.24 ± 4.89	27.5 ± 4.69	<0.001
Obesity, *n* (%)	59 (38.6)	79 (25.5)	0.004
Fasting glucose (mmol/L)	10.21 ± 3.47	5.23 ± 0.7	<0.001
Systolic BP (mmHg)	144.02 ± 17.66	150.72 ± 29.79	0.01
Diastolic BP (mmHg)	88.53 ± 9.63	88.34 ± 14.06	0.883
Pulse BP (mmHg)	55.49 ± 13.16	62.38 ± 21.33	<0.001
Mean BP (mmHg)	107.03 ± 11.28	109.14 ± 18.07	0.187
Arterial hypertension, *n* (%)	107 (69.9)	156 (50.2)	<0.001
Current smokers, *n* (%)	50 (32.7)	91 (29.3)	0.452

Categorical variables compared by *χ*^2^ test and continuous variables by *t*-test. T2DM: type 2 diabetes mellitus; BMI: body mass index; BP: blood pressure.

**Table 3:** Genotype and allele distribution in comparison groups.

	T2DM (*n* = 153)		Control (*n* = 311)	
	*n*	%	*n*	%
Genotypes				
TT	101	66	184	59.2
TC	40	26.2	111	35.7
CC	12	7.8	16	5.1
*P*	0.087			
Alleles				
T	242	79.1	479	77
C	64	20.9	143	23
*P*	0.475			
*P*_HWE_	0.009		0.888	

T2DM: type 2 diabetes mellitus; *P*_HWE_: *P* value for the Hardy-Weinberg equilibrium.

**Table 4:** Association analysis between *BGLAP Hind*III and T2DM development.

Model	*P*_c_	OR_c_ (95% CI)	*P*_a_	OR_a_ (95% CI)	*P*_a_^b^
Dominant	0.155	0.746 (0.498-1.117)	0.169	0.745 (0.489-1.134)	0.676
Recessive	0.254	1.569 (0.723-3.406)	0.134	1.861 (0.826-4.191)	0.536
Overdominant	0.04	0.638 (0.415-0.979)	0.03	0.608 (0.389-0.952)	0.12
Additive^a^	0.058	0.656 (0.425-1.015)	0.05	0.635 (0.403-1.0)	0.2
	0.437	1.366 (0.622-3.001)	0.26	1.606 (0.704-3.663)	1

*P*_c_: crude *P* value; OR_c_: crude odds ratio; *P*_a_: *P* value after adjustment for age, sex, BMI, smoking, and arterial hypertension presence; OR_a_: adjusted odds ratio; *P*_a_^b^: *P* value after Bonferroni correction. ^a^Upper row describes the comparison between CT and TT genotypes and lower row between CC and TT genotypes.

**Table 5:** Association analysis between *BGLAP Hind*III and T2DM development among obese and nonobese individuals.

Regression model^1^		*P*_c_	OR_c_ (95% CI)	*P*_c_^int^	*P*_a_	OR_a_ (95% CI)	*P*_a_^int^	*P* ^b^	*P* ^intb^
Dominant		0.011	0.387 (0.186-0.806)	0.029	0.018	0.399 (0.187-0.852)	0.046	0.072	0.184
		0.889	1.036 (0.634-1.693)		0.969	1.01 (0.609-1.675)		1	

Recessive		0.221	4.179 (0.424-41.224)	0.419	0.193	4.868 (0.449-52.757)	0.402	0.772	1
		0.338	1.525 (0.643-3.616)		0.277	1.641 (0.672-4.01)		1	

Overdominant		0.003	0.305 (0.141-0.661)	0.023	0.004	0.308 (0.138-0.687)	0.038	0.016	0.152
		0.692	0.9 (0.534-1.516)		0.575	0.858 (0.501-1.467)		1	

Additive	CT vs. TT	0.004	0.318 (0.146-0.693)	0.024	0.006	0.319 (0.142-0.717)	0.037	0.024	0.148
		0.828	0.943 (0.553-1.605)		0.722	0.905 (0.522-1.568)		1	
	CC vs. TT	0.37	2.864 (0.286-28.629)	0.605	0.293	3.581 (0.332-38.672)	0.527	1	1
		0.372	1.495 (0.619-3.61)		0.331	1.574 (0.631-3.928)		1	

^1^Upper row represents the results for individuals with BMI ≥ 30 kg/m^2^ and lower row for those with BMI < 30 kg/m^2^. *P*_c_: crude *P* value; *P*_c_^int^: crude *P* value for interactive term; *P*_a_: *P* value adjusted for age, sex, smoking, and arterial hypertension presence; *P*_a_^int^: *P* value adjusted for age, sex, smoking, and arterial hypertension presence for interaction term; *P*^b^: *P* value adjusted for Bonferroni correction; *P*^intb^: *P* value adjusted for Bonferroni correction for interaction term.

**Table 6:** Association analysis between *BGLAP Hind*III and T2DM development among males and females.

Regression model^1^	*P*_c_	OR_c_ (95% CI)	*P*_c_^int^	*P*_a_	OR_a_ (95% CI)	*P*_a_^int^	*P* ^b^	*P* ^intb^
Dominant	0.406	0.794 (0.461-1.368)	0.705	0.511	0.829 (0.474-1.449)	0.594	1	1
	0.216	0.678 (0.366-1.254)		0.198	0.658 (0.348-1.245)		0.792	

Recessive	0.734	1.208 (0.406-3.596)	0.509	0.491	1.776 (0.336-9.405)	0.499	1	1
	0.227	2.079 (0.634-6.822)		0.128	2.63 (0.756-9.147)		0.512	

Overdominant	0.304	0.741 (0.419-1.312)	0.428	0.313	0.739 (0.411-1.33)	0.338	1	1
	0.053	0.521 (0.269-1.008)		0.033	0.476 (0.241-0.941)		0.132	

Additive								
CT vs. TT	0.324	0.747 (0.419-1.333)	0.495	0.365	0.759 (0.418-1.379)	0.387	1	1
	0.08	0.549 (0.281-1.073)		0.055	0.506 (0.252-1.014)		0.22	
CC vs. TT	0.871	1.096 (0.363-3.314)	0.596	0.556	1.409 (0.45-4.412)	0.555	1	1
	0.385	1.708 (0.511-5.711)		0.187	2.351 (0.66-8.377)		0.748	

^1^Upper row represents the results for males and lower row for females. *P*_c_: crude *P* value; *P*_c_^int^: crude *P* value for interactive term; *P*_a_: *P* value adjusted for age, sex, smoking and arterial hypertension; *P*_a_^int^: *P* value adjusted for age, sex, smoking and arterial hypertension for interaction term; *P*^b^: *P* value adjusted for Bonferroni correction; *P*^intb^: *P* value adjusted for Bonferroni correction for interaction term.

**Table 7:** Clinical characteristics of T2DM patients stratified by *BGLAP Hind*III genotypes.

Characteristics	Genotype			*P*
	TT (*n* = 101)	TC (*n* = 40)	CC (*n* = 12)	
BMI (kg/m^2^)	29.59 ± 4.9	28.55 ± 5.26	28.56 ± 3.45	0.460
Fasting glucose (mmol/L)	10.23 ± 3.59	10.26 ± 3.33	9.87 ± 3.07	0.938
HbA1c (%)	8.51 ± 2.7	8.87 ± 2.57	8.25 ± 2.89	0.694
Total cholesterol (mmol/L)	5.47 ± 1.29	5.16 ± 1.12	5.14 ± 1.26	0.331
HDL cholesterol (mmol/L)	0.957 ± 0.27	0.898 ± 0.31	0.992 ± 0.33	0.452
LDL cholesterol (mmol/L)	3.47 ± 1.26	3.28 ± 1.14	3.35 ± 1.35	0.699
Triglyceride (mmol/L)	1.94 ± 1.76	1.88 ± 0.66	1.63 ± 0.66	0.795
Systolic BP (mmHg)	143.76 ± 18.85	146.13 ± 15.63	139.17 ± 13.11	0.476
Diastolic BP (mmHg)	88.61 ± 10.1	88.38 ± 8.65	88.33 ± 9.37	0.989
Pulse BP (mmHg)	55.15 ± 13.68	57.75 ± 13.25	50.83 ± 5.15	0.254
Mean BP (mmHg)	107 ± 12.04	107.63 ± 9.61	105.28 ± 10.49	0.820

*n*: number of cases; BMI: body mass index, HDL: high-density lipoprotein; LDL: low-density lipoprotein; BP: blood pressure.

## Data Availability

The data used to support the findings of this study are available from the corresponding author upon request.
